# Update on How to Approach a Patient with Locked-In Syndrome and Their Communication Ability

**DOI:** 10.3390/brainsci14010092

**Published:** 2024-01-17

**Authors:** Kaitlyn Voity, Tara Lopez, Jessie P. Chan, Brian D. Greenwald

**Affiliations:** 1 Hackensack Meridian School of Medicine, Nutley, NJ 07110, USA; kaitlyn.voity@hmhn.org; 2 JFK Johnson Rehabilitation Institute, Edison, NJ 08820, USA; taralopz@gmail.com (T.L.); jessie.chan@hmhn.org (J.P.C.)

**Keywords:** locked-in syndrome, communication after stroke, locked-in syndrome rehabilitation, establishing communication, defects following locked-in syndrome

## Abstract

Locked-in syndrome (LIS) is a rare and challenging condition that results in tetraplegia and cranial nerve paralysis while maintaining consciousness and variable cognitive function. Once acute management is completed, it is important to work with the patient on developing a plan to maintain and improve their quality of life (QOL). A key component towards increasing or maintaining QOL within this population involves the establishment of a functional communication system. Evaluating cognition in patients with LIS is vital for evaluating patients’ communication needs along with physical rehabilitation to maximize their QOL. In the past decade or so, there has been an increase in research surrounding brain–computer interfaces to improve communication abilities for paralyzed patients. This article provides an update on the available technology and the protocol for finding the best way for patients with this condition to communicate. This article aims to increase knowledge of how to enhance and manage communication among LIS patients.

## 1. Introduction

LIS was first identified as severe tetraplegia, cranial nerve paralysis, and anarthria, but with persevering awareness and ocular movements. The prevalence and incidence of LIS are not well understood, which is likely due to a combination of rarity as well as common misdiagnosis. A survey of Dutch nursing homes in 2013 found a prevalence of LIS in 0.7 per 10,000 residents [1]. In Norway, all hospitals are required to refer LIS patients to the Norwegian National Unit for Rehabilitation of LIS, therefore creating a comprehensive database. A study utilizing this registry found that in 2021, 51 out of Norway’s population of 5.5 million people had complete or incomplete LIS lasting more than 6 weeks [2].

The pathophysiology behind LIS is a vascular injury to the brain stem, specifically the ventral pons or ventral midbrain, while the cerebrum, for the most part, remains unaffected. This type of injury is traditionally caused by ischemia or a hemorrhagic infarction [2,3]. However, traumatic brain injury can also elicit this syndrome; the mechanism of injury is typically blunt or penetrating trauma, leading to an insult to the vertebrobasilar artery blood supply [2].

Classical LIS is described as quadriplegia and anarthria with preserved awareness and vertical eye movements [3]. This constellation of symptoms results from any injury to the ventral pons or the caudal ventral midbrain while the cerebrum remains unaffected [2]. 

LIS has three main subtypes based on impairments, which vary based on the nature of the offending lesion, but retention of consciousness is present in all subtypes. Isolated conjugate vertical eye movements are pathognomonic for classical LIS and result from lesions affecting structures, as detailed in Table 1. Incomplete LIS is characterized by the addition of voluntary movements or eye movements aside from conjugate up-gaze. Total LIS is described as total immobility, including loss of vertical eye movements. This literature review will focus on communication methods for patients with classical LIS, but it is worth noting that impairments exist on a spectrum based on the nature of the injury and stage of recovery [3].

**Figure 1:**
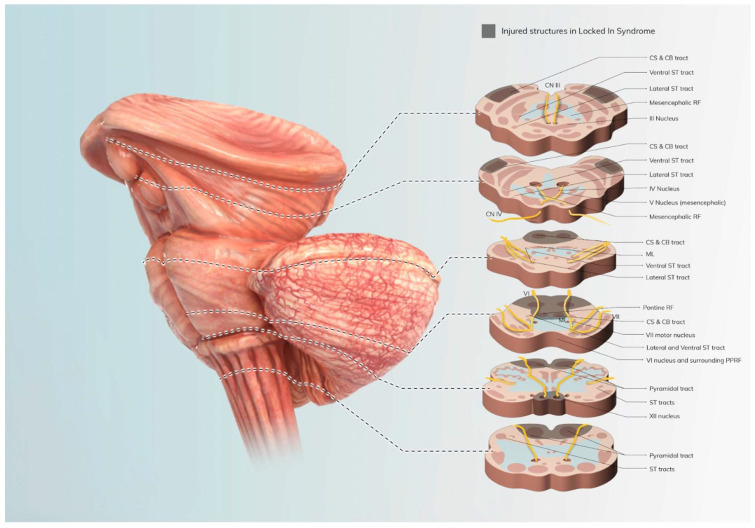
Pathophysiology of LIS. This image shows the structures damaged in LIS, including CS: corticospinal track; CB: corticobulbar track; ST: spinothalamic track; RF: reticular formation; ML: medical lemniscus; PPRF: paramedian pontine reticular formation.

While arousal and awareness are preserved in LIS, cognitive function is variably impacted by the lesion, as shown in Figure 1. Cognitive function is most impacted immediately post-injury, and impairments in attention, memory, and cognitive endurance are commonly observed acutely [3]. Observational studies have suggested that LIS patients recover reading and oral comprehension, visual recognition, spatial orientation, right–left discrimination, short-term memory, and intellectual functioning [4]. Many of the patients, however, were found to have residual impairments in complex sentence comprehension, mental calculation, problem solving, working memory, mental flexibility, distractibility, impulse control, and executive functioning [4]. Rousseaux et al. speculated that the impairments in higher-level cognitive functioning could be secondary to the cerebellar lesion, which may not have initially been isolated to the ventral pons or resulted in impaired cerebral blood flow. Environmental causes should not be overlooked, as LIS patients often experience chronic low mental and physical stimulation as well as social isolation, which impact cognitive functioning and predispose patients to depression, which can present with attention and memory deficits [4]. 

LIS is a profoundly challenging condition that significantly impacts an individual’s QOL. Characterized by complete paralysis of the body’s voluntary muscles while cognitive functions remain intact, locked-in syndrome severely limits communication and physical movement. Patients with this condition can often feel extreme isolation from this condition as they are unable to communicate and have a lack of movement [5]. Not only can this take a toll on the patient, but also on their loved ones. LIS cannot be cured, but there are ways to improve the patient’s QOL. As stated before, patients with LIS are usually able to maintain eye movement and preserve cognitive function. A major milestone in an improvement in QOL for these individuals is utilizing the eye gaze as a potential way to communicate. It is extremely important that, in an acute setting, a baseline of the patient’s communication ability be explored and appropriately evaluated.

## 2. Materials and Methods

**Figure 2:**
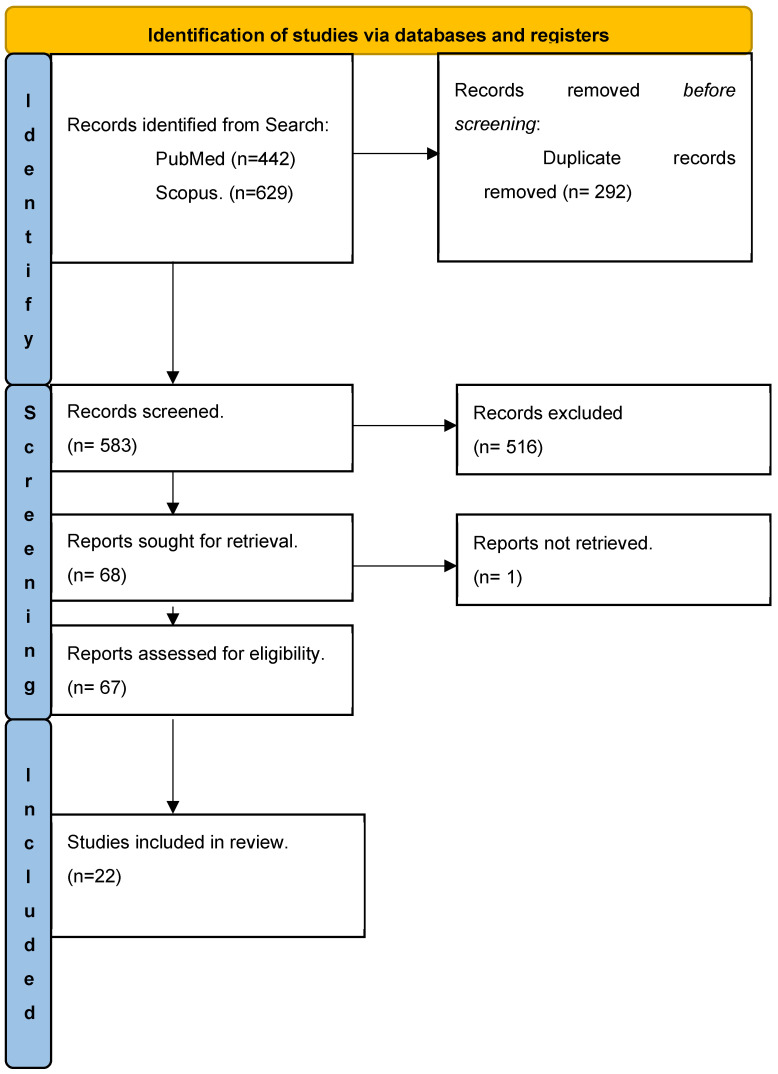
Process flow diagram.

A literature review was conducted using the databases PubMed and Scopus, covering the period between 2013 and 2023. The search terms used were “Locked-in Syndrome”, “augmentative alternative communication”, “locked in syndrome rehabilitation”, “no technology augmentative alternative communication (AAC)”, “LOW TECH AAC”, and “HIGH TECH AAC”. The Boolean operator “AND” was employed to refine the search and obtain relevant results. After the initial search, articles not written in English, published before 2013, irrelevant to the topic, and in duplicate were excluded. In total, we found 292 papers related to the research topic. A total of 203 were excluded due to the criteria listed above. We found 88 papers to use in our review, as seen in Figure 2. Additionally, three articles were not identified through the original online dataset search but from speech-language pathology articles identified through PubMed.

### Data Reporting

Descriptive information was extracted from the articles, including the author, country, and year of publication. We analyzed the study type, the means of communication expressed in the articles, and the etiology of the syndrome. Information regarding the different systems of communication used was then grouped into invasive BCI vs. non-invasive BCI to highlight key features.

## 3. Establishing Communication

One of the first steps in this process is confirming the level of consciousness. This involves the observation of patients’ behaviors and responses to stimuli in order to determine their ability to follow commands and establish the reliability of their yes/no response. It is important to note any premorbid conditions that may affect an individual’s ability to communicate, including age. Once a reliable communication means is obtained or established, the evolution of more intricate and advanced communication methods ranging from no-tech to high-tech augmentative and alternative communication (AAC) can be introduced and explored [6]. However, due to limited or minimal residual muscle activity as a result of LIS, traditional augmentative and alternative communication methods may not be sufficient. Therefore, a brain–computer interface (BCI) could provide patients with LIS the ability to communicate with the use of solely neural signals versus requiring the use of muscle activity [7]. 

### 3.1. Overview of Solutions for Communication

**Figure 3:**
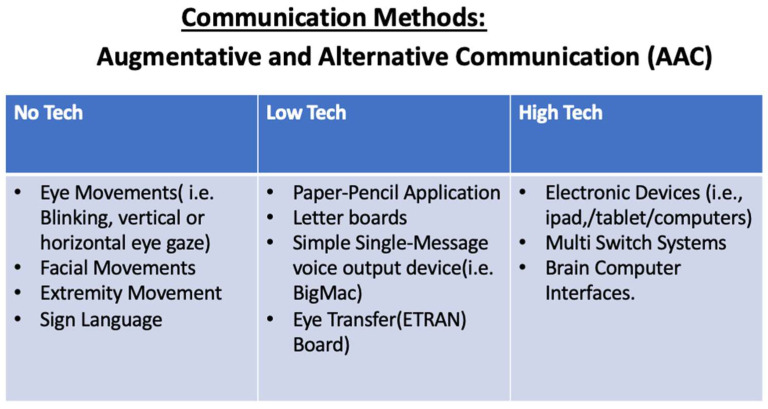
Communication methods.

Communication methods can range from no-tech, low-tech, or high-tech augmentative and alternative communication, as seen in Figure 3. No-tech does not require the use of additional materials. Communication is solely obtained through the use of bodily movements such as eye movements (i.e., blinking, looking up–down, or right–left), sign language, facial movements, or extremity movements on command, etc. [6]. When no-tech communication methods are utilized, specific movements have a specific communicative purpose. During communication exchanges, both communication partners must be aware of the individual’s methods and means of communication.

Based on the individual’s eye movement and cognitive function, low-tech AAC can be explored. Low-tech AAC can involve the use of materials such as paper and pencil, letter boards, or simple single-message voice output devices (i.e., BigMac) in addition to oneself to functionally communicate. [6]. When an individual with LIS has preserved ocular movements, the use of eye gaze with letter boards (i.e., Eye Transfer (ETRAN) Board or EyeLink Board) can be an effective way to communicate. This is conducted by selecting single letters or clusters of letters via fixation or personal means of selection (i.e., blinking) [8]. 

Lastly, a more advanced communication method is the use of high-tech AAC. High-tech AAC involves the use of technology to aid in functional communication. This can include external communication devices such as tablets or computers [6]. For individuals with LIS, eye-gaze switches, eye tracking, or eye-gaze devices can be utilized in order to control electronic devices (i.e., tablet/computers) to communicate functionally. In addition to the use of eye-gaze devices, an additional access method includes BCI [9]. BCI allows patients with LIS to communicate by utilizing neural signals in conjunction with current high-tech AAC devices and programs [9]. 

### 3.2. Brain–Computer Interface (BCI) 

#### Overview

**Figure 4:**
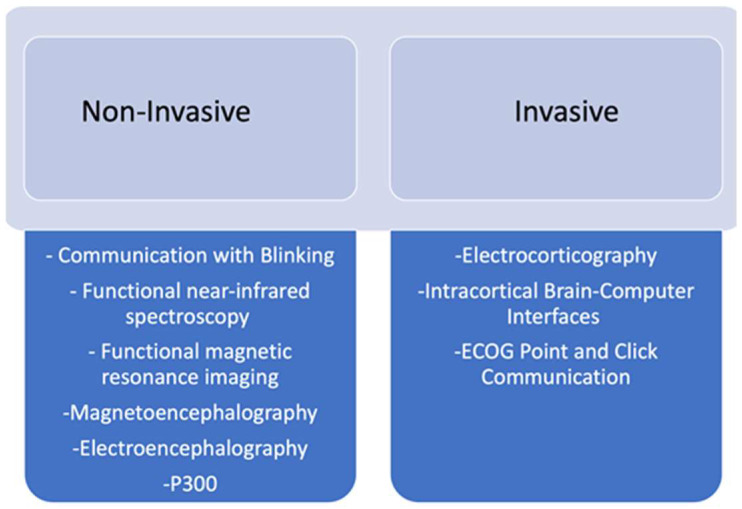
Invasive vs. non-invasive.

BCIs involve the use of neural activity instead of muscle activity to aid in functional communication. Once raw neural signals are obtained via invasive or non-invasive means, the BCI can identify and decode signals in order to synthesize a functional message [10]. Examples of a non-invasive BCI would be communication with blinking. BCIs allow individuals with severe motor and cognitive defects to improve their QOL [11]. The goal of this review is to discuss the current research and development on the topic of BCIs and examine the risks and benefits associated with non-invasive vs. invasive methods. In Figure 4, we list the different types of Invasive vs Noninvasive. It is also important to discuss the useability, accessibility, and patient satisfaction associated with these means of communication. 

### 3.3. Non-Invasive BCIs

LIS presents a profound challenge where individuals retain cognitive function but are almost entirely paralyzed and unable to communicate using conventional means. In recent years, neuroscience and technology have advanced significantly, giving rise to innovative solutions aimed at improving the lives of those affected by LIS. One such avenue of exploration is the development of non-invasive BCIs, which hold the promise of establishing a direct link between brain activity and external devices. These BCIs enable the translation of the users’ neurological signals into meaningful commands, potentially allowing them to communicate, control devices, and interact with their environment. In Table 2, we list the specific sources used and what information we took from the article.

#### 3.3.1. Communication through Blinking

Communicating through blinking is a crucial method for individuals with LIS. Ocular movements such as blinking or specific eye movements (i.e., up/down, right/left) become a lifeline, enabling individuals to convey their thoughts, needs, and desires [12]. When communicating with ocular movements, there is a variety of AAC with different styles and capabilities ranging from blinking, the use of transparent panels with numbers and/or letters (i.e., Eye Transfer Boards), eye-gaze sensing screens, eye tracking systems, and the use of modified switches to control different applications presented on devices [8]. 

Typically, it has been found that within the LIS population, there are preserved voluntary movements including eye blinking and vertical eye movements. When communicating, an example of the use of eye blinking is when an individual blinks once for no and twice for yes. Another common example is prolonged eye for yes [13]. Although simplistic, eye movement can be an essential method of communication [13]. While establishing an effective communication modality, it is important to keep in mind that all communication methods must be individualized to suit the strengths and reliability of the individual. Using a system of blinks and eye movements complemented by caregivers and/or devices, individuals with LIS can spell out words, select options on communication boards, and answer yes-or-no questions. This method, while slow compared to typical communication, empowers individuals with LIS to bridge the gap between their inner thoughts and the outside world, providing them with a means to express themselves and maintain vital connections with their loved ones and caregivers. It exemplifies the remarkable resilience and adaptability of the human spirit in the face of profound physical limitations.

In addition to blinking, external switches in conjunction with a switch interface can be an effective way to communicate. With this method, the switch interface receives input and directly relays it to the AAC/program/computer/toy to create the intended message or action. External switches can be attached to external wearable items, such as glasses, to be activated with the use of blinking [13]. 

More complex AAC involves eye-gaze technology such as screen-based eye sensors, eye tracking systems, and modified external switches to control applications on devices. Eye-gaze programs may require an individual to make a selection as the program systematically scans different potential icons and messages (i.e., when the system is scanning, items could be highlighted or bolded when toggling from icon to icon). An additional method may require the individual to blink when gazing at their target in order to make a selection [13]. Eye tracking software integrated with speech-generating devices such as Tobii Dynavox can provide individuals with an effective form of communication.

#### 3.3.2. Electroencephalography (EEG)

Electroencephalography (EEG) is a valuable tool for assessing brain activity and communication possibilities by recording electrical activity along the scalp, providing insights into brain function. Despite its relatively lower spatial resolution compared to other imaging techniques, EEG offers high temporal resolutions, making it suitable for real-time communication applications [10]. BCIs that utilize EEG signals have been developed to enable locked-in individuals to control external devices, spell words, or answer yes-or-no questions by modulating their brainwave patterns. While the reliability and accuracy of EEG-based BCIs can be influenced by factors like signal noise and individual variability, ongoing advancements in signal processing algorithms and hardware design enhance performance. These developments hold promise for improving the QOL for individuals with severe motor disabilities, highlighting the potential of EEG-based BCIs in the fields of neuro communication and assistive technology. 

#### 3.3.3. Emerging Technologies 

##### Functional Near-Infrared Spectroscopy

Functional near-infrared spectroscopy (fNIRS) is a non-invasive tool that uses near-infrared light to monitor brain oxygen levels, helping to assess brain activity in patients with LIS. It detects brain activity in specific regions associated with mental tasks, enabling patients to answer questions or spell words using deliberate neural activations [14]. However, its effectiveness depends on the individual’s condition and responsiveness, as well as their ability to use the technology. fNIRS monitors levels of oxygenated hemoglobin, which indicate increased brain activity in the region [11]. fNIRS has emerged as a promising tool for communication; however, more research is needed prior to clinical application.

##### Functional Magnetic Resonance Imaging

Functional magnetic resonance imaging (fMRI) is a neuroimaging technique that has gained substantial recognition in LIS research. This technology operates by harnessing robust magnetic fields and radio waves to gauge alterations in blood flow, providing valuable insight into brain activity [16]. In the context of LIS, fMRI may have immense potential as a means of facilitating communication when used in conjunction with a BCI. It discerns unique patterns of brain activity associated with various cognitive tasks, thereby empowering individuals to express their intentions and responses effectively. Nevertheless, it is worth noting that the efficacy of fMRI with BCI as a communication tool can be influenced by the individual’s capacity to consistently generate distinct brain activation patterns. Furthermore, fMRI with BCI necessitates a highly controlled environment, which may hinder its portability and accessibility for real-time communication applications [11]. As researchers continue to explore the capabilities of fMRI with BCI as a communication tool to advance QOL, it is imperative to consider these limitations.

##### Magnetoencephalography 

Magnetoencephalography (MEG) is a neuroimaging technique that records the magnetic fields generated by neuronal activity, offering exceptional temporal resolution and precise localization of brain regions engaged in diverse cognitive processes. MEG’s unique capability to capture rapid neural dynamics makes it highly valuable in the context of detecting intention and communication efforts among individuals with LIS. This is achieved through an analysis of the timing and spatial distribution of neural signals, allowing researchers to discern patterns linked to specific mental tasks or responses [11]. However, the practical application of MEG is constrained by factors such as susceptibility to movement artifact and the requirement for specialized equipment and controlled environments. Despite these limitations, MEG holds significant promise as a tool for advancing our understanding of brain function in LIS and potentially facilitating communication strategies for affected individuals.

##### P300 

The P300 event-related potential, a distinctive neural response occurring after the presentation of a relevant stimulus, represents a promising avenue for enhancing communication among individuals with LIS. With this method, individuals are exposed to visual or auditory stimuli, and their brains generate a P300 waveform when they recognize a specific target stimulus, such as a name or designated word [15]. The P300-based BCI approach offers an efficient and non-intrusive channel for communication among locked-in individuals. By directing their attention towards the desired option, they can trigger a detectable P300 response, which can be monitored using EEG or other neuroimaging techniques. While this approach necessitates intact cognitive abilities and sustained attention, it circumvents the need for precise motor control, rendering it particularly well suited for individuals with limited physical mobility.

##### Summary of Risks and Benefits of Non-Invasive BCIs 

Non-invasive BCIs face the challenge of detecting and interpreting weak and variable neural signals, which can result in potential inaccuracies in translating intention into action. This necessitates calibration and training periods that demand time and patience from users. Moreover, the usability and effectiveness of non-invasive BCIs exhibit substantial variability contingent upon an individual’s cognitive and physical capabilities, potentially restricting their applicability to specific subsets of locked-in patients. Nevertheless, the upside of non-invasive BCIs is their immense potential to significantly enhance QOL. These interfaces empower users by allowing them to spell words, make selections, and manipulate objects solely through cognitive processes, which allow for improved communication and autonomy. This can mitigate feelings of isolation and frustration, thereby promoting emotional well-being and fostering a heightened sense of agency. Striking a balance between these potential risks and rewards necessitates a continuous cycle of innovation, close collaboration among researchers, clinicians, and patients, and consideration of each individual’s unique circumstances and requirements.

### 3.4. Invasive 

Invasive BCIs involve the implantation of electrodes directly into the brain and offer a remarkable avenue for establishing a direct and precise link between neural signals and devices. In Table 3, we list the specific sources used and what information we took from the article.

#### 3.4.1. Electrocorticography

Electrocorticography (ECoG) emerges as a promising neuroimaging technique with substantial potential for communication. EcoG involves the strategic placement of electrodes directly on the brain’s surface, affording the capability to record electrical activity with heightened spatial and temporal precision compared to non-invasive methodologies. This advantage translates into a more refined ability to pinpoint neural activity patterns associated with intention and cognition, facilitating the identification of specific brain regions and their activations. Consequently, EcoG holds the promise of substantially enhancing the accuracy of decoding an individual’s thoughts and intentions. However, the utilization of EcoG in the context of LIS requires careful consideration. The procedure necessitates the surgical implantation of electrodes, which introduces potential risks and mandates meticulous monitoring and post-operative care. Additionally, the interpretation of EcoG signals is inherently complex and necessitates the application of advanced signal processing techniques to distill meaningful information. Nonetheless, there exists optimism in the realm of machine learning and computational methods, offering avenues for improving the accuracy and usability of EcoG-based BCIs.

##### ECOG Point and Click Communication 

In this study, participants who were already enrolled in the Brain Gate program, which involves the implantation of electrodes, were examined to investigate the differences between a standard QWERTY keyboard and one developed by Brain Gate. The development known as Electrocorticography Point and Click Communication represents a noteworthy advancement within the domain of BCIs designed specifically for individuals with locked-in syndrome. This innovative approach capitalizes on the exceptional spatial and temporal resolution capabilities of EcoG to enable users to exert control over external devices through their brain signals, effectively translating their cognitive intentions into actionable commands [17]. The EcoG Point and Click Communication paradigm entails the placement of electrode arrays directly onto the surface of the brain, facilitating the detection of neural patterns linked to specific intentions, such as maneuvering a cursor to select items on a screen. The precision afforded by EcoG technology empowers users to manipulate a cursor with remarkable accuracy, enabling a point-and-click interaction akin to the traditional usage of computer mice. By concentrating their attention on various on-screen options, users can elicit distinct neural responses, which are subsequently decoded by sophisticated algorithms to initiate desired actions. This method equips individuals with the ability to engage in tasks such as word composition, message creation, or navigation of computer interfaces without necessitating physical movement, thus representing a significant leap forward in assistive technology for the LIS population.

#### 3.4.2. Intracortical

Intracortical BCIs epitomize a cutting-edge and invasive approach with substantial potential to empower individuals with LIS. This technique involves the surgical implantation of electrodes directly into the cerebral cortex, affording the capability to meticulously and instantaneously record neural activity [17]. This technology harbors the potential to establish a direct conduit between cognition and external devices, thereby enabling communication and the manipulation of surroundings. The intracortical BCI method provides an unparalleled level of spatial and temporal resolution, facilitating the detection of specific neural activity patterns associated with distinct intentions [18]. This granular level of control opens up avenues for more sophisticated forms of communication, including intricate speech generation and the dexterous operation of robotic prosthetics closely mirroring natural limb movements. Such precision holds the promise of substantially elevating QOL for individuals with LIS, endowing them with a notable degree of agency and independence.

##### Summary of Risks and Benefits of Invasive BCI’s 

Invasive BCIs present both remarkable benefits and notable risks in the context of locked-in syndrome. With the unique combination of intact cognition and severe motor impairment, locked-in syndrome necessitates innovative solutions to bridge the gap between the mind and the body. Invasive BCIs involve the implantation of electrodes directly into the brain, offering a direct and precise link between neural signals and devices. Through the placement of electrodes on the brain’s surface, EcoG provides heightened spatial and temporal precision, enhancing the accuracy of decoding an individual’s thoughts and intentions. This approach can facilitate communication, interaction with technology, and the restoration of a degree of agency and independence. However, the surgical implantation of EcoG electrodes introduces potential medical risks and necessitates meticulous post-operative care.

Intracortical BCIs, on the other hand, offer unparalleled spatial and temporal resolution by implanting electrodes directly into the cerebral cortex. This technology allows for the detection of specific neural activity patterns, offering the potential for intricate forms of communication and precise control over external devices. While this precision can significantly enhance the QOL of individuals with LIS, the procedure is highly invasive, posing surgical risks and raising ethical considerations.

In conclusion, the use of invasive and intracortical BCIs in the context of LIS carries the potential for groundbreaking benefits, including improved communication and autonomy. However, the invasive nature of these procedures introduces medical risks and ethical complexities. The decision to pursue invasive BCIs should be made on a case-by-case basis, considering the individual’s specific circumstances and weighing the potential benefits against the inherent risks. Continued research and technological advancements are essential to optimize the safety and effectiveness of these approaches.

## 4. Limitations: Affordability, Usability, Access, Grants, Insurance, Social Worker 

Despite the ability of BCIs to potentially decode neural signals to synthesize functional messages, there are a variety of limitations that can negatively impact the success and reliability of the device. These limitations can include usability, efficiency, reliability, access, and cost. Clinicians must also advocate for patients to obtain the right level of assistance.

According to Elliot et al., individuals who utilized BCIs for communication exhibited slower and less accurate responses with decreased usability compared to individuals who utilized different AAC methods (i.e., eye-tracking cameras and EyeLink board). It was found that BCI required a higher cognitive workload compared to the use of an eye-tracking camera and the EyeLink board. As a result of these findings, it was found that BCI was not superior in reference to measures of speed, workload, and usability compared to other AAC means [19]. In addition, due to the emotional and cognitive involvement required for individuals and caregivers to learn and navigate BCI, individuals often resort to different communication systems in order to communicate faster [20].

Currently, within the field, there is a limited standardized clinical pathway regarding formal assessment, selection, administration, and funding for clinical use of BCI devices [20]. A lack of standardization and consistency between AAC research and BCI procedures has negatively impacted the implementation of BCI in a functional setting [20,21]. Additional limitations, such as increased setup time, slow speed of use, and the requirement of technology support for caregivers, result in limited implementation of BCI systems as primary means of communication outside of the research setting [19]. Pitt et al. found that clinicians utilizing AAC have limited interest in the use of BCIs in clinical settings and have reported poor reliability with BCIs. In addition, the increased time and knowledge for setup required to utilize the device negatively and impact useability [20]. 

## 5. Caregiver Burden 

Caring for an individual with LIS can yield a variety of new challenges. Caregivers can often be impacted by a variety of stressors, such as limited or lack of communication with the patient, limited support from loved ones, a large burden of daily responsibilities, a lack of resources for support and information, financial stressors, role change, or isolation [22]. Of note, Zinkevich et al. found that caregiver burden is significantly reduced with the implementation of successful AAC interventions.

## 6. Conclusions

LIS is a rare and debilitating condition characterized by severe motor impairment while retaining cognitive function and awareness. The pathophysiology involves brainstem injury, often due to vascular complications or traumatic brain injury. The presentation of LIS can vary, with classical LIS being the most well-known subtype, characterized by quadriplegia, anarthria, and preserved vertical eye movements. Cognitive impairments can vary but are often observed, affecting attention, memory, and higher-level cognitive functions. Communication is a critical aspect of improving the QOL of individuals with LIS, and various invasive and non-invasive methods have been explored. Non-invasive BCIs offer communication methods with fewer risks but can present challenges related to signal detection and interpretation, usability, and standardization. Invasive BCIs, such as ECoG and intracortical interfaces, provide a more direct and precise connection between neural signals and external devices, offering the potential for advanced communication and control. While BCIs hold great potential, the use of BCIs is limited in terms of affordability, usability, access, and caregiver burden. The fields of AAC and BCI research are evolving rapidly, and ongoing efforts are needed to address these limitations and make these technologies more accessible and effective for individuals with LIS. In caring for individuals with LIS, it is crucial to consider not only the technological advancements but also the emotional and social aspects of their lives. Caregiver burden can be significant, but the implementation of AAC interventions has shown promise in reducing this burden and improving the overall QOL for both patients and caregivers. By continuing research and innovation in communication technologies, there is hope for enhancing the lives of those affected by LIS and providing them with the means to engage more fully with the world around them.

## Figures and Tables

**Table 1:** Structure involved in LIS.

Structure (s)	Function	Status in Classical LIS
CN 3, 4	Vertical eye movement, pupillary reflex, eyelid control data	Intact
CN 6, paramedian pontine reticular formation	Bilateral horizontal gaze	Injured
Corticobulbar tracts (CN 5, 7, 9, 10, 11, 12)	Respiratory function	Variable
Corticospinal tracts	Limb and truncal motor functions	Injured
Medial lemniscus and spinothalamic pathways	Sensation	Intact
Reticular activating system	Arousal, consciousness, awareness	Intact

**Table 2:** Non-invasive BCI references.

Reference	Communication Involved
Kopsky et al. (2014) (Disabil Rehabil, 36(20), 1723–1727, doi:10.3109/09638288.2013.866700) [12].	Through utilizing the maintained eye movement in LIS, a specialized spelling system was tried to see if it helped make communication with blinking more user-friendly.
Park et al. (2012) (Ann Rehabil Med, 36(2), 268–272, doi:10.5535/arm.2012.36.2.268) [13].	After eye blinking therapy (AAC), there was a benefit seen in patients’ communication ability.
Luo et al. (2022) (Neurother J Am Soc Exp Neurother, 19(1), 263–273, doi:10.1007/s13311-022-01190-2) [10].	With the use of EEG, brain activity was able to be tracked before and during communication to see the areas most involved.
Vansteensel et al. (2022) (Neurorehabil Neural Repair, 36(10–11), 666–677, doi:10.1177/15459683221125788) [11].	fNRI and fMRI are both emerging technologies in the area of communication, but the one downside is implanting them in daily life. They allow for the tracking and amplification of neural signals.
Butler et al. (2020) (Am J Speech Lang Pathol, 29(3), 1674–1701, doi:10.1044/2020_AJSLP-19-00050) [14].	fNRI may not only help with augmenting communication but also show promise in assisting with diagnosing communication ability.
Lugo et al. (2016) (Front Hum Neurosci, 10, 569, doi:10.3389/fnhum.2016.00569) [15]	P300 allows for better diagnosis in baseline communication ability for patients with LIS while looking at event potentials when presented with a task or stimuli.

**Table 3:** Invasive BCI references.

Reference	Communication Involved
Bacher et al. (2015) (Neurorehabil Neural Repair, 29(5), 462–471, doi:10.1177/1545968314554624) [17].	In the clinical trial of the BrainGate Neural Interface System, this system, featuring the BrainGate Radial Keyboard, was compared to a standard QWERTY keyboard. The results showed a significant improvement in communication ability. The participant effectively used this interface for face-to-face communication with research staff through text-to-speech conversion and remote communication via an internet chat application.
Homer et al. (2013) (Annu Rev Biomed Eng, 15, 383–405, doi:10.1146/annurev-bioeng-071910-124640) [18].	Central components of intracortical BCIs include implanted sensors capturing neural signals and the decoding software extracting the user’s intended movements from these signals. These innovations can enhance the technology’s capacity, precision, and durability.

## References

[1] Kohnen R.F., Lavrijsen J.C.M., Bor J.H.J., Koopmans R.T.C.M. (2013). The prevalence and characteristics of patients with classic locked-in syndrome in Dutch nursing homes. J. Neurol..

[2] M Das J., Anosike K., Asuncion R.M.D. (2023). Locked-in Syndrome. StatPearls.

[3] Farr E., Altonji K., Harvey R.L. (2021). Locked-In Syndrome: Practical Rehabilitation Management. PMR.

[4] Rousseau M.-C., Baumstarck K., Alessandrini M., Blandin V., Billette de Villemeur T., Auquier P. (2015). Quality of life in patients with locked-in syndrome: Evolution over a 6-year period. Orphanet J. Rare Dis..

[5] Corallo F., Bonanno L., Buono V.L., De Salvo S., Rifici C., Pollicino P., Allone C., Palmeri R., Todaro A., Alagna A. (2017). Augmentative and Alternative Communication Effects on Quality of Life in Patients with Locked-in Syndrome and Their Caregivers. J. Stroke Cerebrovasc. Dis..

[6] Elsahar Y., Hu S., Bouazza-Marouf K., Kerr D., Mansor A. (2019). Augmentative and Alternative Communication (AAC) Advances: A Review of Configurations for Individuals with a Speech Disability. Sensors.

[7] Vansteensel M.J., Jarosiewicz B. (2020). Brain-computer interfaces for communication. Handbook of Clinical Neurology.

[8] Ezzat M., Maged M., Gamal Y., Adel M., Alrahmawy M., El-Metwally S. (2023). Blink-To-Live eye-based communication system for users with speech impairments. Sci. Rep..

[9] Brumberg J.S., Pitt K.M., Mantie-Kozlowski A., Burnison J.D. (2018). Brain–Computer Interfaces for Augmentative and Alternative Communication: A Tutorial. Am. J. Speech-Lang. Pathol..

[10] Luo S., Rabbani Q., Crone N.E. (2022). Brain-Computer Interface: Applications to Speech Decoding and Synthesis to Augment Communication. Neurotherapeutics.

[11] Vansteensel M.J., Branco M.P., Leinders S., Freudenburg Z.F., Schippers A., Geukes S.H., Gaytant M.A., Gosselaar P.H., Aarnoutse E.J., Ramsey N.F. (2022). Methodological Recommendations for Studies on the Daily Life Implementation of Implantable Communication-Brain–Computer Interfaces for Individuals with Locked-in Syndrome. Neurorehabilit. Neural Repair.

[12] Kopsky D.J., Winninghoff Y., Winninghoff A.C.M., Stolwijk-Swüste J.M. (2014). A novel spelling system for locked-in syndrome patients using only eye contact. Disabil. Rehabil..

[13] Park S.-W., Yim Y.-L., Yi S.-H., Kim H.-Y., Jung S.-M. (2012). Augmentative and Alternative Communication Training Using Eye Blink Switch for Locked-in Syndrome Patient. Ann. Rehabil. Med..

[14] Butler L.K., Kiran S., Tager-Flusberg H. (2020). Functional Near-Infrared Spectroscopy in the Study of Speech and Language Impairment Across the Life Span: A Systematic Review. Am. J. Speech-Lang. Pathol..

[15] Lugo Z.R., Quitadamo L.R., Bianchi L., Pellas F., Veser S., Lesenfants D., Real R.G.L., Herbert C., Guger C., Kotchoubey B. (2016). Cognitive Processing in Non-Communicative Patients: What Can Event-Related Potentials Tell Us?. Front. Hum. Neurosci..

[16] Vansteensel M.J., Pels E.G.M., Bleichner M.G., Branco M.P., Denison T., Freudenburg Z.V., Gosselaar P., Leinders S., Ottens T.H., Boom M.A.V.D. (2016). Fully Implanted Brain–Computer Interface in a Locked-In Patient with ALS. N. Engl. J. Med..

[17] Bacher D., Jarosiewicz B., Masse N.Y., Stavisky S.D., Simeral J.D., Newell K., Oakley E.M., Cash S.S., Friehs G., Hochberg L.R. (2015). Neural Point-and-Click Communication by a Person With Incomplete Locked-In Syndrome. Neurorehabilit. Neural Repair.

[18] Homer M.L., Nurmikko A.V., Donoghue J.P., Hochberg L.R. (2013). Sensors and Decoding for Intracortical Brain Computer Interfaces. Annu. Rev. Biomed. Eng..

[19] Elliott C., Sutherland D., Gerhard D., Theys C. (2022). An Evaluation of the P300 Brain–Computer Interface, EyeLink Board, and Eye-Tracking Camera as Augmentative and Alternative Communication Devices. J. Speech Lang. Hear. Res..

[20] Pitt K.M., Brumberg J.S. (2022). Evaluating person-centered factors associated with brain–computer interface access to a commercial augmentative and alternative communication paradigm. Assist. Technol..

[21] Pitt K.M., Brumberg J.S., Pitt A.R. (2019). Considering Augmentative and Alternative Communication Research for Brain-Computer Interface Practice. Assist. Technol. Outcomes Benefits.

[22] Zinkevich A., Uthoff S.A.K., Wirtz M.A., Boenisch J., Sachse S.K., Bernasconi T., Feldhaus M., Ansmann L. (2022). Burden of informal caregivers of people without natural speech: A mixed-methods intervention study. BMC Health Serv. Res..

